# Enteral Nutrition and Hydration in Patients with Acute Stroke: Efficacy of an Automatic Pump System for Water Administration and Flushes—A Pilot Study

**DOI:** 10.3390/s22208029

**Published:** 2022-10-20

**Authors:** Alex Buoite Stella, Paolo Manganotti

**Affiliations:** Clinical Unit of Neurology, Department of Medicine, Surgery and Health Sciences, University Hospital and Health Services of Trieste–ASUGI, University of Trieste, Strada di Fiume, 447, 34149 Trieste, Italy

**Keywords:** artificial nutrition, dysphagia, cerebrovascular diseases, hydration

## Abstract

Background: Enteral nutrition is often prescribed in acute stroke to meet energy and fluid needs in patients with dysphagia. Tubes clogging represent a common complication of enteral formula delivery, requiring substitution and influencing nutrition administration. Frequent water flushes are recommended as one of the most effective procedures to prevent tube occlusion, but it might be time demanding and not consistently performed by the healthcare staff. This study aimed to assess the efficacy of an automatic flush pump, compared to a manual flush system, to prevent tubes’ occlusions in acute-stroke patients, as this might affect nutrition and hydration. Methods: Gastrointestinal symptoms, nutrition and hydration biomarkers were also monitored to determine the different devices’ safety. Sixty-two patients were included in the study and allocated to the “manual” or “automatic” flushes device. Results: The mean duration of data collection was 7 ± 2 days. Tube occlusions occurred in 22.6% of the patients in the “manual” group, whereas only one tube clogging was reported in the “automatic” group (*p* = 0.023). No significant differences between groups were reported for constipation and diarrhea frequency nor nutrition and hydration status. When the nurses were asked to simulate manual flush administration at the same frequency of the automatic device, they were able to meet the recommendations only 10% of the time. Conclusion: This preliminary study suggests the efficacy of automatic flush systems to prevent enteral tube clogging, without affecting health status compared to standard manual flush systems.

## 1. Introduction

Enteral nutrition is necessary to provide nutritional and fluid support in patients who are not able to meet their nutritional requirements through oral intake alone. Most of these patients are characterized by neurological diseases, such as stroke, which might result in dysphagia [1]. Malnutrition may be present in patients with stroke in the acute care setting, showing a prevalence between 8% and 34%, and was associated with higher mortality rates, reduced functional capacity and reduced quality of life at 6 months after stroke [2]. Hydration status in stroke patients was studied only in the last decade, with results suggesting dehydration may be associated with worse discharge outcomes [3,4,5,6]. In addition, though dysphagia might not be associated with poor nutrition status during the first week after stroke, it might induce a higher risk of dehydration [7].

Recent guidelines for nutritional support in acute-care patients with stroke highlighted the importance of a proper evaluation, both at admission and during hospitalization, to treat and prevent malnutrition, electrolytes and fluid unbalance [8,9,10]. When proper oral nutrition is not possible, such as in patients with severe dysphagia, artificial nutrition is suggested. Enteral nutrition should be preferred and based on national and international guidelines, it is delivered with the insertion of a nasogastric tube (NGT) in the first 2–3 weeks of artificial nutrition and with the insertion of a percutaneous endoscopic gastrostomy (PEG) for artificial nutrition of longer duration (more than 4–6 weeks). The importance of a proper nutritional support, if necessary with enteral or parenteral nutrition, has also been highlighted in other international guidelines for patients’ best care (National Institute of Health and Care Excellence) [11], suggesting the required levels of nutrients, calories and fluids. Preliminary results on a sample of individuals with stroke in acute care receiving enteral nutrition suggested that most of the patients did not reach the minimum required levels for optimal fluid intake of 30–35 mL/kg/day, despite considering the water coming from the enteral formulas and the water that was manually flushed [12].

Delivering nutritional formulas through enteral feeding tubes, both with NGT and PEG, includes the risk of tube occlusion that sometimes requires its removal and substitution if unclogging is not possible. To prevent occlusions, it is necessary to flush tubes with water, even every 6 h and every time before and after drug administration [13]. Nevertheless, manual water flushes require the complete attention of the nurse or caregiver and quite often, the high-demanding tasks of a semi-intensive care unit do not allow enough time for proper flushing protocols. Continuous or “bolus” water administration is considered equally valid as for the most recent guidelines for enteral nutrition [11,14].

The primary aim of this study was to report frequency of tube occlusion during enteral nutrition in an acute setting in patients after stroke, who are fed with enteral nutrition during the Neurology–Stroke Unit acute-care hospitalization. The secondary aim was to evaluate the effects on gastrointestinal symptoms, as well as on nutrition and hydration biomarkers, of a small-boli water administration system compared to a continuous-water-administration protocol. Finally, nurses impressions about the implementation of the automatic flush system were also investigated.

## 2. Materials and Methods

An observational descriptive prospective study was performed by collecting data from a sample of patients who were admitted with an acute stroke diagnosis in a hub-center stroke unit (ASUGI, Trieste) between March 2020 and March 2021. Inclusion criteria were: patients of both sexes, of any age, with a clinical diagnosis of stroke confirmed by neuroimaging (computed tomography, CT, or magnetic resonance imaging, MRI), both ischemic and hemorrhagic, who are prescribed enteral nutrition using an NGT. Additionally, as part of the standard protocol to be treated in the stroke unit, a negative nasopharyngeal swab testing for SARS-CoV-2 was mandatory at admission. Participants were excluded if (i) poor survival chances at 3 days were expected based on the clinical and neuroimaging evaluation (e.g., unfavorable core/penumbra volumes at CT perfusion) [15,16], (ii) duration of the enteral nutrition therapy was expected to be less than 5 days or (iii) if enteral nutrition was started no earlier than 5 days from admission. Informed consent was obtained for each participant and the study was conducted according to the principles of the Declaration of Helsinki, approved by the Institutional Review Board of the University Hospital where the study was performed and by the regional Ethics Committee (CEUR FVG 214/2019).

Participants received enteral nutrition through i) a standard “one-way” pump which requires manual water administration and flushes (manual) (Flocare Infinity, Danone Nutricia, Netherlands) and ii) a “two-way” pump (one for nutrition and one for water), with automatic water administration and flushes (automatic) (Kangaroo e-pump system, Cardinal Health, Ireland). The type of pump was randomly assigned depending on the stroke unit room where patients were admitted. Clinical and care practices in the stroke unit were uniform and did not differ between the different in-patient rooms. As such, the neurologist prescribed nutrition and fluid therapy based on standard care and protocols, without any difference based on the type of enteral pump used (“manual” or “automatic”). The only difference was the rate of water administration: in the “manual” group, patients received water continuously for a 12 h period (i.e., water was continuously administered with a minimal flow) and flushes were performed manually by the nurse after therapy administration; conversely, patients allocated in the “automatic” group received water through small automatic boli every 1–2 h during the same 12 h period. No difference was present between the type and composition of the nutritional formulas administered. To evaluate nurses’ ability to provide water boli even with a standard one-way pump, they were asked to put a signature (“mimicking” the activity of providing the bolus manually) on a specific time chart collocated near the bed of a subsample of 20 patients. To reduce the bias risk arising from the inability to conduct a blinded study, all healthcare professionals were asked to strictly comply with standard practice and therapy was prescribed following standard protocols, which are independent of the type of pump. During the enteral nutrition period and for a maximum of 10 days, daily data were collected about enteral nutrition complications, such as tube occlusions and substitutions, or altered gastrointestinal function (constipation and diarrhea). In addition, during the same period, daily data were collected about the therapy, the volume of enteral formulas and fluids administered (eventually integrated with oral or parenteral nutrition). Nutrition and hydration assessments were performed at admission and at discharge. Nutrition status was assessed with the Mini Nutritional Assessment tool (MNA) [17] and by measuring mid-upper arm circumference (MUAC) [18]. MNA and circumference measurements were provided by the same operator with the same tape measure. Blood and urinary samples were collected as part of standard practice and urinary and serum osmolality (uOsm and sOsm) were analyzed as biomarkers of hydration status.

A survey was designed and administered to the nurses to investigate their impressions of the implementation of the automatic flush system, by administering the survey before and after the study. Survey questions asked about their knowledge of the differences in water administration systems, the time needed to administer water boli, the possibility of providing frequent water flushes, main NGT complications and the expected beneficial effects of the automatic water administration system. A second survey was administered after the study to assess nurses’ satisfaction.

### Statistical Analysis

Descriptive epidemiology and demographics were used to describe the sample, presenting means and standard deviations (sds) or proportions. A within–between ANOVA test was performed to assess differences between groups and between the admission and discharge time of measurement for all nutrition and hydration biomarkers. The greenhouse–Geisser correction was applied to adjust for lack of sphericity. Bonferroni correction was selected for post hoc analysis. According to score classification, MNA changes between admission and discharge were rated as worsening, no change or improvement. Chi-square and independent *t*-test were used to compare outcomes between the two groups. The significance level was set at *p* < 0.05.

## 3. Results

During the study period, 424 patients were admitted at the stroke unit with an acute-stroke diagnosis; among these, 76 received a diagnosis of dysphagia and were prescribed enteral nutrition therapy. Eleven of them were not included in the study due to a poor short-term prognosis, while three were excluded as the duration of the enteral nutrition therapy was expected to be less than 5 days. As such, 62 patients were included in the study and final analysis. The mean duration of data collection was 7 ± 2 days. No significant differences were found in demographic and anamnestic characteristics between the “manual” and the “automatic” groups (Table 1). Length of stay was not possible to be considered as an outcome due to healthcare system reorganization during the COVID-19 pandemic, resulting in some patients staying in the stroke unit for less or more time than usually needed, depending on daily organizational needs. A total of 209 and 214 cumulative days of enteral nutrition were investigated in the “manual” and “automatic” group, respectively.

### 3.1. NGT Occlusions and Gastrointestinal Symptoms

Occlusions of the NGT were reported in seven (22.6%) participants in the manual group and in one (3.2%) in the automatic group (*p* = 0.023). During the duration of the study, tube occlusions happened only once, 4 ± 2 days after starting enteral nutrition, without new occlusions after the first tube substitution. Constipation was not reported in any of the participants. Diarrhea was present in 38.7% of the manual group, with 2 ± 1 days of diarrhea for each patient and 32.3% of the automatic group, with 2 ± 1 days of diarrhea for each patient, without differences between groups (*p* = 0.596 and *p* = 1.000, respectively, for prevalence and duration). Results are shown in Table 2.

### 3.2. Nutrition and Hydration Biomarkers

Comparison of the middle-upper arm circumference at admission and at discharge between the “manual” and the “automatic” group showed a significant effect for time (*p* < 0.001), with no significant effect for group (*p* = 0.442) or time*group (*p* = 0.972). Post hoc analysis revealed a significant decrease in the circumference of 0.17 ± 0.23 cm in the manual (*p* < 0.001) and 0.21 ± 0.18 cm in the automatic group (*p* < 0.001). Total fluids (including intravenous and those received through EN) were similar between groups (*p* = 0.898). Comparison of the hydration biomarkers at admission and at discharge between the “manual” and the “automatic” group showed, for uOsm, a significant effect for time (*p* < 0.001), with no significant effect for group (*p* = 0.465) or time*group (*p* = 0.821), while no differences were found for sOsm for time (*p* = 0.581), group (*p*= 0.224) or time*group (*p* = 0.830). Post hoc analysis revealed a significant increase in uOsm of 30 ± 53 mOsm in the manual (*p* = 0.007) and 27 ± 48 mOsm in the automatic group (*p* = 0.008). Results are shown in Table 3.

### 3.3. Nurses’ Satisfaction and Compliance

Results from the survey indicated that before the implementation of the automatic flush system, 86.7% of the nurses were not aware of the potential differences in water administration systems, 93.7% complained about the time needed to provide water boli, 67.9% referred to rarely being able to provide frequent manual flushes, 63.5% reported tube occlusions as one of the main complications and 51.2% were doubtful about the efficacy of the automatic flush system. After the study, none of the nurses was unsatisfied with the use of the automatic flush system, 15.5% reported no improvement in their working habits and 84.5% reported their working habits improved after the implementation of the automatic flush system. Pilot testing suggested that nurses were able to provide manual boli at the same frequency as the automatic flush system only in 10% of the cases.

## 4. Discussion

From this study, the prevalence of dysphagia or patients requiring artificial nutrition was found to be 17.9%, which is in line with previous investigations [19,20]. Enteral feeding pumps are commonly used in the clinical setting and at patients’ homes, with different types of devices being developed to better promote adherence and safety [21]. Enteral tube clogging remains one of the main challenges to artificial nutrition and different solutions to reduce the risk of occlusions have been suggested, including warm water flushes, enzyme treatments and mechanical occlusion clearing devices [22]. Automatic tube flush systems have been developed to administer water boli at preset volumes and frequency [23], to reduce the risk of enteral tube occlusions without requiring frequent manual flushes. Indeed, frequent water flushes are recommended, with a minimum volume of 30 mL of water every 4 h during continuous feedings or before and after intermittent feedings [24,25,26]. Nevertheless, such practice is time demanding and preliminary observations, published on a poster, suggested that only 34.8% of the ordered flushes can effectively be administered when only manual administration is possible [27]. In this study, when nurses were asked to mimic manual flushing at the same frequency as the automatic flush system, they were able to simulate water administration in only 10% of the prescribed procedures.

Despite the large use of pumps for enteral nutrition delivery, few studies have investigated the efficacy of different types of devices in preventing tube clogging. In addition, current guidelines suggest that delivering water continuously or at small boli should have similar effects on patients’ recovery and safety [24]. Results from this study suggest that the automatic flush system was able to prevent the risk of tube clogging, as only one occlusion was reported in 1 out of 31 patients in the automatic flushing group, for a total of 214 days of enteral nutrition, whereas seven events of NGT occlusion were reported in the manual flushing group, over a total of 209 days. These results confirm preliminary observations reporting no occlusion in an automatic flush system compared to 57% of tube occlusion in a manual flush system [28]. Safety of automatic water boli administration was assessed by the prevalence of gastrointestinal symptoms after enteral nutrition started. From this study, no significant differences were reported between groups in the rate of constipation or diarrhea. In acute-stroke patients, enteral tube feeding is usually associated with a diarrhea frequency of up to 27.7% compared to those not requiring enteral nutrition (6.2%) and this might lead to a higher risk of malnutrition, dehydration and altered electrolytes and acid-base balance [29]. Higher prevalence has been reported in other studies in critically ill patients receiving enteral nutrition [30]. In this study, at least one episode of diarrhea was reported in 38.7% and 32.3% of the patients in the manual and automatic groups, respectively, without significant differences. Nutritional status was assessed by using a validated tool, the MNA and the MUAC, as an anthropometric measure. Both methods have been previously used in stroke patients [31,32]. A previous study on elderly stroke patients found that 54.3% of the participants were malnourished, 37.1% were classified as at risk and 8.6% were well nourished [33]. Results from the present investigation suggested similar proportions at admission, with a tendency to remain stable or worsen at discharge. This finding could be explained by a previous study, showing that many patients requiring enteral nutrition might not receive enough energy intake during the early phase of hospitalization after acute stroke [12]. Similarly, hydration status evaluated on the basis of uOsm and sOsm was found to be indicative of underhydration in most of the participants in both groups, without significant differences between the enteral pumps. Hydration status has been suggested to be associated with clinical and functional outcomes and despite it perhaps primarily depending on hydration parameters at admission [6,34,35], fluid balance during the acute phase of hospitalization might also influence post-stroke recovery [5,34,35].

Enteral nutrition is recognized as an important component in the recovery process and health maintenance of acute-stroke patients; nevertheless, most of the nurses who participated in the survey subjectively reported poor knowledge about the different protocols and devices available and some of them were not informed about the presence of automatic-flush systems. Providing manual flushes at a high frequency is commonly recognized as a highly demanding procedure that can hardly be properly administered with the several tasks of a stroke unit. After the implementation of the automatic-flush pump, most of the nurses were satisfied with the system and suggested it improved their working habits, reducing the risk of water clogging and, therefore, leaving more time for other caring procedures.

Some limitations are present and should be considered when evaluating the results of this study. First of all, the small sample size should be considered and further studies in larger samples are recommended. Nevertheless, the participants were selected with well-defined inclusion and exclusion criteria to be as homogenous as possible to reduce the factors that might have influenced the results. In addition to this, the study was performed during the COVID-19 pandemic, which resulted in a reorganization of the health services and admissions to the different units [36]. Some other measures might have been taken to assess long-term health outcomes (such as the length of stay, enteral nutrition duration or health status at 3–6 months); however, due to the pandemic, such factors might have been affected by the health service reorganization (e.g., follow-up visits were sometimes not performed or performed in a short phone interview). Despite this study only representing an exploratory and pilot investigation conducted on a small sample of patients and only in those with a favorable prognosis, these findings encourage the efficacy of automatic water administration pumps for enteral nutrition to reduce the risk of tube clogging compared to manual-flush systems, without any difference in safety outcomes as gastrointestinal symptoms, nutrition or hydration biomarkers. Since the high proportion of acute-stroke patients requiring enteral nutrition and the potential complications due to tube occlusions (e.g., the substitution of the tube, inconsistency of water and energy administration, etc.), an automatic-flush system could be recommended, especially when the healthcare personnel is not able to provide frequent manual flushes.

## 5. Conclusions

Tube clogging can happen in about one-third of cases and can interfere with the proper administration of nutrition formulas and, in some cases, require the substitution of the tubes with discomfort for the patient, an economic cost for the healthcare system and a cost in terms of time for the healthcare professionals. Results from this preliminary study suggest that automatic-flush pumps could help to reduce the risk of enteral tube occlusions without affecting patients’ health and recovery.

## Figures and Tables

**Table 1:** Demographics and clinical characteristics of the included acute-stroke patients (n = 62) in the manual and automatic flushes pumps groups. Means ± standard deviations and proportions.

	Manual n = 31	Automatic n = 31	Significance
Age (y)	72 ± 8	71 ± 7	0.546
Males/Females [n (%)]	16/15 (52/48)	18/13 (58/42)	0.609
Body Mass (kg)	75.1 ± 10.2	74.1 ± 7.6	0.673
BMI (kg/m^2^)	25.9 ± 2.3	25.5 ± 2.0	0.497
Ischemic/Hemorrhagic [n (%)]	29/2 (94/6)	28/3 (90/10)	0.640
tPA [n (%)]	20 (65)	21 (68)	0.788
NIHSS	10 ± 3	11 ± 3	0.209
HTN [n (%)]	17 (55)	19 (61)	0.606
DM [n (%)]	9 (29)	10 (32)	0.782
Dyslipidemia [n (%)]	3 (10)	4 (13)	0.688
AF [n (%)]	3 (10)	3 (10)	1.000
Days on enteral nutrition (days)	6 ± 2	7 ± 2	0.053

Notes: BMI, body mass index; tPA, tissue plasminogen activator; NIHSS, National Institute of Health Stroke Scale; HTN, hypertension; DM, diabetes mellitus; AF, atrial fibrillation. Days on enteral nutrition within the study period. Significance for between groups independent samples *t*-test and chi square.

**Table 2:** Gastrointestinal symptoms of the included acute-stroke patients (n = 62) in the manual and automatic flush pump groups. Means ± standard deviations and proportions.

	Manual n = 31	Automatic n = 31	Significance
NGT occlusions [n (%)]	7 (22.6)	1 (3.2)	**0.023**
More than 1 occlusion [n (%)]	0 (0)	0 (0)	1.000
Constipation [n (%)]	0 (0)	0 (0)	1.000
Diarrhea [n (%)]	12 (38.7)	10 (32.3)	0.596
Duration of diarrhea (days)	2 ± 1	2 ± 1	1.000

Notes: NGT, nasogastric tubes. Significance for between groups independent samples *t*-test and chi-square. Bold values for *p* < 0.05.

**Table 3:** Nutrition and hydration biomarkers of the included acute-stroke patients (n = 62) in the manual and automatic flushes pumps groups. Means ± standard deviations and proportions.

	Manual n = 31	Automatic n = 31	Significance
*Nutritional status*			
MNA at admission [n (%)]			0.863
Malnutrition	13 (41.9)	14 (45.2)	
At risk	13 (41.9)	11 (35.5)	
Well-nourished	5 (15.8)	7 (19.3)	
MNA at discharge [n (%)]			0.673
Worsened	6 (18.9)	4 (12.9)	
Remained stable	22 (71.1)	25 (80.6)	
Improved	3 (10.0)	2 (6.5)	
MUAC at admission (cm)	28.3 ± 1.3	28.4 ± 1.4	0.925
MUAC at discharge (cm)	28.2 ± 1.2	28.1 ± 1.4	0.981
*Hydration*			
Total fluid intake (mL/day)	1934 ± 412	1921 ± 387	0.898
uOsm at admission (mOsm)	580 ± 131	559 ± 127	0.522
uOsm at discharge (mOsm)	610 ± 113	586 ± 120	0.423
sOsm at admission (mOsm)	296 ± 5	298 ± 5	0.229
sOsm at discharge (mOsm)	296 ± 5	297 ± 5	0.261

Notes: MNA, Mini Nutritional Assessment; MUAC, mid-upper arm circumference; uOsm, urine osmolality; sOsm, serum osmolality. Significance for between groups independent samples *t*-test and chi-square.

## Data Availability

Anonymized data are available upon reasonable request to the corresponding author, according to the institutional regulations on data protection in clinical studies.
